# British Railway Coaches

**Published:** 1892-06

**Authors:** 


### BRITISH RAILWAY COACHES.

Nothing is more remarkable than the persistence with which Europeans cling to their miserable, little, old-fashioned, clattering railway carriages, except, indeed, the fact that the average unprotected female of the period ventures to travel by herself in such a vehicle. We have come to expect nearly every week the story of some outrage committed in the privacy of such carriages, and yet the railway "guards" continue to shut up together a strange man and a strange woman in a close compartment, as deliberately as if the chief object of their official existence were to furnish sensations to the newspapers and to the trans-Atlantic cables.

As a rule, the European mind, while it remains in Europe, does not propagate new ideas. It has to be transplanted to America to be quickened and enlivened, and taught to step out of old traditionary ruts. The European builds his railway car with seats for six passengers, because his father did ; and, as European trains do not run as full of passengers as American trains do, a great many of the little cars contain only a traveler or two, who rattle along over the country, quite cut off from the observation of the rest of mankind, and as well hidden as though in a tight box on wheels.

Our large, commodious and comfortable railway carriages are the surprise and admiration of the adventurous European, when he condescends to come to this country to make money, or marry some woman who has got money, and he sometimes actually praises our American comforts and conveniences when he returns to his own. But, bless you! they would never think of copying any thing American over there! In our American cars there is no danger of any woman being insulted or wronged ; all around her are men who will suffer no woman to be aggrieved, even by an impertinent look, and at the door is a brakeman who can offer or summon any service in an instant. Nothing but the inevitable stolidity and self-sufficiency of the European railroad managers hinders them from offering similar guarantees of protection to their own passengers. Nothing but the influence of habit seems to prevent the European traveling public from demanding them.

